# OptiClust, an Improved Method for Assigning Amplicon-Based Sequence Data to Operational Taxonomic Units

**DOI:** 10.1128/mSphereDirect.00073-17

**Published:** 2017-03-08

**Authors:** Sarah L. Westcott, Patrick D. Schloss

**Affiliations:** Department of Microbiology and Immunology, University of Michigan, Ann Arbor, Michigan, USA; University of Wisconsin; Roslin Institute, University of Edinburgh; Gladstone Institutes

**Keywords:** 16S rRNA gene, bioinformatics, microbial ecology, microbiome

## Abstract

Assignment of 16S rRNA gene sequences to operational taxonomic units (OTUs) is a computational bottleneck in the process of analyzing microbial communities. Although this has been an active area of research, it has been difficult to overcome the time and memory demands while improving the quality of the OTU assignments. Here, we developed a new OTU assignment algorithm that iteratively reassigns sequences to new OTUs to optimize the Matthews correlation coefficient (MCC), a measure of the quality of OTU assignments. To assess the new algorithm, OptiClust, we compared it to 10 other algorithms using 16S rRNA gene sequences from two simulated and four natural communities. Using the OptiClust algorithm, the MCC values averaged 15.2 and 16.5% higher than the OTUs generated when we used the average neighbor and distance-based greedy clustering with VSEARCH, respectively. Furthermore, on average, OptiClust was 94.6 times faster than the average neighbor algorithm and just as fast as distance-based greedy clustering with VSEARCH. An empirical analysis of the efficiency of the algorithms showed that the time and memory required to perform the algorithm scaled quadratically with the number of unique sequences in the data set. The significant improvement in the quality of the OTU assignments over previously existing methods will significantly enhance downstream analysis by limiting the splitting of similar sequences into separate OTUs and merging of dissimilar sequences into the same OTU. The development of the OptiClust algorithm represents a significant advance that is likely to have numerous other applications.

**IMPORTANCE** The analysis of microbial communities from diverse environments using 16S rRNA gene sequencing has expanded our knowledge of the biogeography of microorganisms. An important step in this analysis is the assignment of sequences into taxonomic groups based on their similarity to sequences in a database or based on their similarity to each other, irrespective of a database. In this study, we present a new algorithm for the latter approach. The algorithm, OptiClust, seeks to optimize a metric of assignment quality by shuffling sequences between taxonomic groups. We found that OptiClust produces more robust assignments and does so in a rapid and memory-efficient manner. This advance will allow for a more robust analysis of microbial communities and the factors that shape them.

**Podcast**: A podcast concerning this article is available.

## Abstract (precis)

The analysis of microbial communities from diverse environments using 16S rRNA gene sequencing has expanded our knowledge of the biogeography of microorganisms. An important step in this analysis is the assignment of sequences into taxonomic groups based on their similarity to sequences in a database or based on their similarity to each other, irrespective of a database. In this study, we present a new algorithm for the latter approach. The algorithm, OptiClust, seeks to optimize a metric of assignment quality by shuffling sequences between taxonomic groups. We found that OptiClust produces more robust assignments and does so in a rapid and memory-efficient manner. This advance will allow for a more robust analysis of microbial communities and the factors that shape them.

## INTRODUCTION

Amplicon-based sequencing has provided incredible insights into Earth’s microbial biodiversity (1, 2). It has become common for studies to include sequencing millions of 16S rRNA gene sequences across hundreds of samples (3, 4). This is a sequencing depth 3 to 4 orders of magnitude greater than was previously achieved using Sanger sequencing (5, 6). The increased sequencing depth has revealed novel taxonomic diversity that is not adequately represented in reference databases (1, 3). However, the advance has forced reengineering of methods to overcome the rate- and memory-limiting steps in computational pipelines that process raw sequences through the generation of tables containing the number of sequences in different taxa for each sample (7–10). A critical component of these pipelines has been the assignment of amplicon sequences to taxonomic units that are defined either based on similarity to a reference or operationally based on the similarity of the sequences to each other within the data set (11, 12).

A growing number of algorithms have been developed to cluster sequences into operational taxonomic units (OTUs). These algorithms can be classified into three general categories. The first category of algorithms has been termed closed reference or phylotyping (13, 14). Sequences are compared to a reference collection and clustered based on the reference sequences to which they are similar. This approach is fast; however, the method struggles when a sequence is similar to multiple reference sequences that may have different taxonomies and when it is not similar to sequences in the reference (15). The second category of algorithms has been called *de novo* because they assign sequences to OTUs without the use of a reference (14). These include hierarchical algorithms such as nearest, furthest, and average neighbor (16) and algorithms that employ heuristics such as abundance- or distance-based greedy clustering (AGC or DGC, respectively) as implemented in USEARCH (17) or VSEARCH (18), Sumaclust and OTUCLUST (19), and Swarm (20). *De novo* methods are agglomerative and tend to be more computationally intense. It has proven difficult to know which method generates the best assignments. A third category of algorithm is open-reference clustering, which is a hybrid approach (3, 14). Here, sequences are assigned to OTUs using closed-reference clustering, and sequences that are not within a threshold of a reference sequence are then clustered using a *de novo* approach. This category blends the strengths and weaknesses of the other method and adds the complication that closed-reference and *de novo* clustering use different OTU definitions. These three categories of algorithms take different approaches to handling large data sets to minimize the time and memory requirements while attempting to assign sequences to meaningful OTUs.

Several metrics have emerged for assessing the quality of OTU assignment algorithms. These have included the time and memory required to run the algorithm (3, 20–22), agreement between OTU assignments and the sequences’ taxonomy (20, 22–32), sensitivity of an algorithm to stochastic processes (33), the number of OTUs generated by the algorithm (23, 34), and the ability to regenerate the assignments made by other algorithms (3, 35). Unfortunately, these methods fail to directly quantify the quality of the OTU assignments. An algorithm may complete with minimal time and memory requirements or generate an idealized number of OTUs, but the composition of the OTUs could be incorrect. These metrics also tend to be subjective. For instance, a method may appear to recapitulate the taxonomy of a synthetic community with known taxonomic structure but do a poor job when applied to real communities with poorly defined taxonomic structure or for sequences that are prone to misclassification. As an alternative, we developed an approach to objectively benchmark the clustering quality of OTU assignments (13, 15, 36). This approach counts the number of true positives (TPs), true negatives (TNs), false positives (FPs), and false negatives (FNs) based on the pairwise distances. Sequence pairs that are within the user-specified threshold and are clustered together represent TPs, and those in different OTUs are FNs. Those sequence pairs that have a distance larger than the threshold and are not clustered in the same OTU are TNs, and those in the same OTU are FPs. These values can be synthesized into a single correlation coefficient, the Matthews correlation coefficient (MCC), which measures the correlation between observed and predicted classifications and is robust to cases where there is an uneven distribution across the confusion matrix (37). Consistently, the average neighbor algorithm was identified as among the best or as the best algorithm. Other hierarchical algorithms, such as furthest and nearest neighbor, which do not permit the formation of FPs or FNs, respectively, fared significantly worse. The distance-based greedy clustering as implemented in VSEARCH has also performed well. The computational resources required to complete the average neighbor algorithm can be significant for large data sets, and so there is a need for an algorithm that efficiently produces consistently high-quality OTU assignments.

These benchmarking efforts have assessed the quality of the clusters after the completion of the algorithm. In the current study, we developed and benchmarked a new *de novo* clustering algorithm that uses real-time calculation of the MCC to direct the progress of the clustering. The result is the OptiClust algorithm, which produces significantly better sequence assignments while making efficient use of computational resources.

## RESULTS

### OptiClust algorithm.

The OptiClust algorithm uses the pairs of sequences that are within a desired threshold of each other (e.g., 0.03), a list of all sequence names in the data set, and the metric that should be used to assess clustering quality. A detailed description of the algorithm is provided for a toy data set in the supplemental material. Briefly, the algorithm starts by placing each sequence either within its own OTU or into a single OTU. The algorithm proceeds by interrogating each sequence and recalculating the metric for the cases where the sequence stays in its current OTU, is moved to each of the other OTUs, or is moved into a new OTU. The location that results in the best clustering quality indicates whether the sequence should remain in its current OTU or be moved to a different or new OTU. Each iteration consists of interrogating every sequence in the data set. Although numerous options are available for optimizing the clusters and for assessing the quality of the clusters within the mothur-based implementation of the algorithm (e.g., sensitivity, specificity, accuracy, F1 score, etc.), the default metric for optimization and assessment is MCC because it includes all four parameters from the confusion matrix (see Fig. S1 and Table S1 in the supplemental material). The algorithm continues until the optimization metric stabilizes or until it reaches a defined stopping criterion.

10.1128/mSphereDirect.00073-17.1 TEXT S1 Worked example of how OptiClust algorithm clusters sequences into OTUs. Download TEXT S1, PDF file, 1.6 MB. Copyright © 2017 Westcott and Schloss. 2017 Westcott and Schloss This content is distributed under the terms of the Creative Commons Attribution 4.0 International license.

10.1128/mSphereDirect.00073-17.2 FIG S1 The OptiClust algorithm is able to effectively cluster sequences into OTUs by minimizing or maximizing numerous metrics. Plot of MCC (A), number of OTUs (B), and execution times (C) for the comparison of output from the OptiClust algorithm when minimizing or maximizing a variety of parameters when applied to four natural and two synthetic data sets. Within mothur, OTU assignments can also be made using other metrics, including minimizing false positives and maximizing the specificity, positive predictive value, and true negatives; however, these all resulted in sequences being assigned to separate OTUs, which resulted in no false positives and the maximum number of true negatives. The error bars indicate the range of values observed for 10 replicates. Download FIG S1, EPS file, 0.8 MB. Copyright © 2017 Westcott and Schloss. 2017 Westcott and Schloss This content is distributed under the terms of the Creative Commons Attribution 4.0 International license.

10.1128/mSphereDirect.00073-17.4 TABLE S1 Summary of the average number of true positives, true negatives, false positives, false negatives, and the resulting Matthews correlation coefficient for each of the clustering methods that were analyzed in this study for each of the six data sets. Blank values indicate that those conditions could not be completed in 50 h with 45 GB of RAM. Download TABLE S1, PDF file, 0.02 MB. Copyright © 2017 Westcott and Schloss. 2017 Westcott and Schloss This content is distributed under the terms of the Creative Commons Attribution 4.0 International license.

### OptiClust-generated OTUs are more robust than those from other methods.

**FIG 1:**
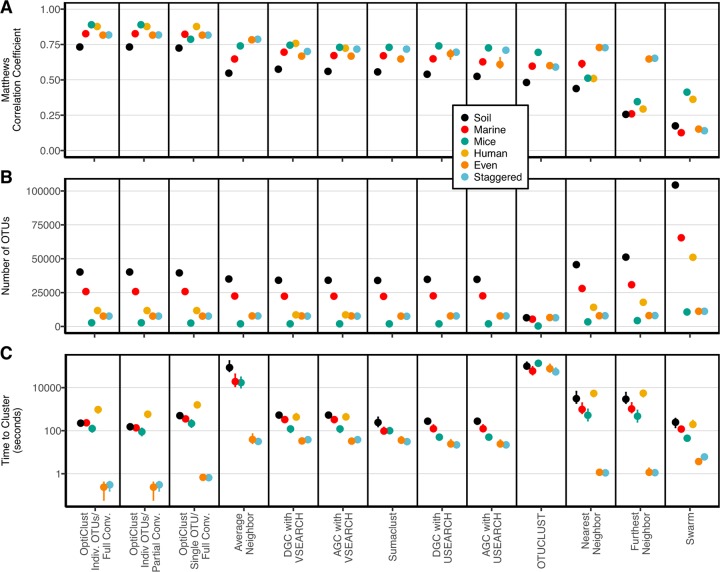
Comparison of *de novo* clustering algorithms. Plot of MCC (A), number of OTUs (B), and execution times (C) for the comparison of *de novo* clustering algorithms when applied to four natural and two synthetic data sets. The first three columns of each panel contain the results of clustering the data sets: (i) seeding the algorithm with one sequence per OTU and allowing the algorithm to proceed until the MCC value no longer changed, (ii) seeding the algorithm with one sequence per OTU and allowing the algorithm to proceed until the MCC changed by less than 0.0001, and (iii) seeding the algorithm with all of the sequences in one OTU and allowing the algorithm to proceed until the MCC value no longer changed. The human data set could not be clustered by the average neighbor, Sumaclust, USEARCH, or OTUCLUST with less than 45 GB of RAM or 50 h of execution time. The median from 10 reorderings of the data is presented for each method and data set. The range of observed values is indicated by the error bars, which are typically smaller than the plotting symbol.

To evaluate the OptiClust algorithm and compare its performance to other algorithms, we utilized six data sets including two synthetic communities and four previously published large data sets generated from soil, marine, human, and murine samples (Table 1). When we seeded the OptiClust algorithm with each sequence in a separate OTU and ran the algorithm until complete convergence, the MCC values averaged 15.2 and 16.5% higher than the OTUs using average neighbor and distance-based greedy clustering (DGC) with VSEARCH, respectively (Fig. 1; Table S1). The number of OTUs formed by the various methods was negatively correlated with their MCC value (ρ = −0.47; *P* < 0.001). The OptiClust algorithm was considerably faster than the hierarchical algorithms and somewhat slower than the heuristic-based algorithms. Across the six data sets, the OptiClust algorithm was 94.6 times faster than average neighbor and just as fast as DGC with VSEARCH. The human data set was a challenge for a number of the algorithms. OTUCLUST and Sumaclust were unable to cluster the human data set in less than 50 h, and the average neighbor algorithm required more than 45 GB of RAM. The USEARCH-based methods were unable to cluster the human data using the 32-bit free version of the software that limits the amount of RAM to approximately 3.5 GB. These data demonstrate that OptiClust generated significantly more robust OTU assignments than existing methods across a diverse collection of data sets with performance that was comparable to popular methods.

**TABLE 1:** Description of data sets used to evaluate the OptiClust algorithm and compare its performance to other algorithms^a^

Data set (reference[s])	Read length (nt)	No. of samples	Total no. of sequences	No. of unique sequences	No. of distances	No. of OTUs
Soil (41)	150	18	948,243	143,677	11,775,167	40,216
Marine (42)	250	7	1,384,988	75,923	12,908,857	25,787
Mice (40)	250	360	2,825,495	32,447	6,988,306	2,658
Human (39)	250	489	20,951,841	121,281	38,544,315	11,648
Even (34, 36)	NA	NA	1,155,800	11,558	29,694	7,651
Staggered (34, 36)	NA	NA	1,156,550	11,558	29,694	7,653

a Each data set contains sequences from the V4 region of the 16S rRNA gene. The number of distances for each data set indicates those that were less than or equal to 0.03. The number of OTUs was determined using the OptiClust algorithm. The even and staggered data sets were generated by extracting the V4 region from full-length reference sequences, and the data sets from the natural communities were generated by sequencing the V4 region using an Illumina MiSeq with paired reads of either 150 or 250 nt. NA, not applicable.

### OptiClust stopping criteria.

By default, the mothur-based implementation of the algorithm stops when the optimization metric changes by less than 0.0001; however, this can be altered by the user. This implementation also allows the user to stop the algorithm if a maximum number of iterations is exceeded. By default, mothur uses a maximum value of 100 iterations. The justification for allowing incomplete convergence was based on the observation that numerous iterations are performed that extend the time required to complete the clustering with minimal improvement in clustering (Fig. S2). We evaluated the results of clustering to partial convergence (i.e., a change in the MCC value that was less than 0.0001) or until complete convergence of the MCC value (i.e., until it did not change between iterations) when seeding the algorithm with each sequence in a separate OTU (Fig. 1). The small difference in MCC values between the output from partial and complete convergence resulted in a difference in the median number of OTUs that ranged between 1.5 and 17.0 OTUs. This represented a difference of less than 0.15%. Among the four natural data sets, between 3 and 6 iterations were needed to achieve partial convergence and between 8 and 12 iterations were needed to reach full convergence. The additional steps required between 1.4 and 1.7 times longer to complete the algorithm. These results suggest that achieving full convergence of the optimization metric adds computational effort; however, considering that full convergence took between 2 and 17 min, the extra effort was relatively small. Although the mothur default setting is partial convergence, the remainder of our analysis used complete convergence to be more conservative.

10.1128/mSphereDirect.00073-17.3 FIG S2 The OptiClust algorithm rapidly converges to optimize the Matthews correlation coefficient. The six data sets were clustered into OTUs using the OptiClust algorithm seeking to maximize the Matthews correlation coefficient. This was repeated 10 times for each data set. Download FIG S2, EPS file, 0.3 MB. Copyright © 2017 Westcott and Schloss. 2017 Westcott and Schloss This content is distributed under the terms of the Creative Commons Attribution 4.0 International license.

### Effect of seeding OTUs on OptiClust performance.

By default, the mothur implementation of the OptiClust algorithm starts with each sequence in a separate OTU. An alternative approach is to start with all of the sequences in a single OTU. We found that the MCC values for clusters generated when seeding OptiClust with the sequences as a single OTU were between 0% and 11.5% lower than when seeding the algorithm with sequences in separate OTUs (Fig. 1). Interestingly, with the exception of the human data set (0.2% more OTUs), the number of OTUs was as much as 7.0% lower (mice) than when the algorithm was seeded with sequences in separate OTUs. Finally, the time required to cluster the data when the algorithm was seeded with a single OTU was between 1.5 and 2.9 times longer than if sequences were seeded as separate OTUs. This analysis demonstrates that seeding the algorithm with sequences as separate OTUs resulted in the best OTU assignments in the shortest period of time.

### OptiClust-generated OTUs are as stable as those from other algorithms.

One concern that many have with *de novo* clustering algorithms is that their output is sensitive to the initial order of the sequences because each algorithm must break ties where a sequence could be assigned to multiple OTUs. An additional concern specific to the OptiClust algorithm is that it may stabilize at a local optimum. To evaluate these concerns, we compared the results obtained using 10 randomizations of the order that sequences were given to the algorithm. The median coefficient of variation across the six data sets for MCC values obtained from the replicate clusterings using OptiClust was 0.1% (Fig. 1). We also measured the coefficient of variation for the number of OTUs across the six data sets for each method. The median coefficient of variation for the number of OTUs generated using OptiClust was 0.1%. Confirming our previous results (15), all of the methods that we tested were stable to stochastic processes. Of the methods that involved randomization, the coefficient of variation for MCC values was considerably smaller with OptiClust than with the other methods, and the coefficient of variation for the number of OTUs was comparable to the other methods. The variation observed in clustering quality suggested that the algorithm does not appear to converge to a locally optimum MCC value. More importantly, the random variation does yield output of a similarly high quality.

### Time and memory required to complete optimization-based clustering scales efficiently.

**FIG 2:**
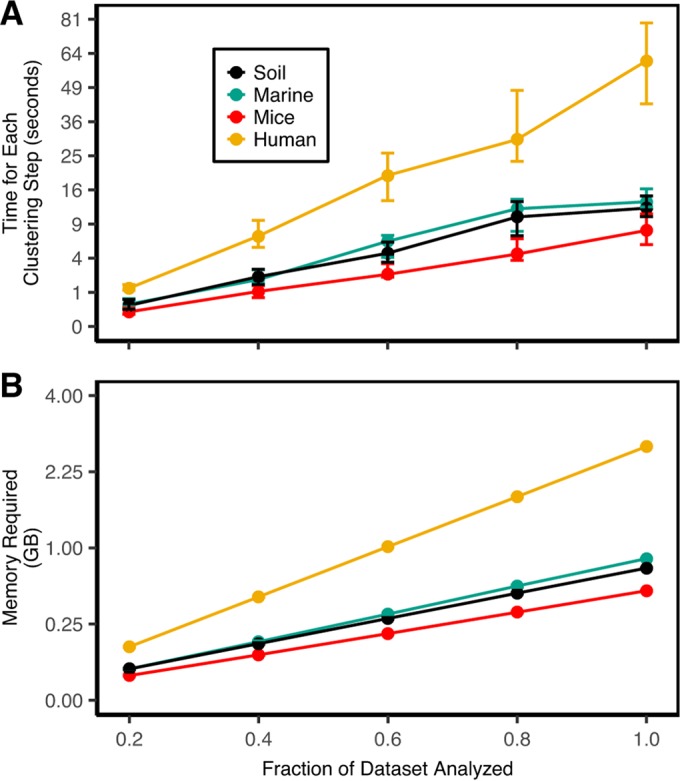
OptiClust performance. Average execution time (A) and memory usage (B) required to cluster the four natural data sets. The confidence intervals indicate the range between the minimum and maximum values. The *y* axis is scaled by the square root to demonstrate the relationship between the time and memory requirements relative to the number of unique sequences squared.

Although not as important as the quality of clustering, the amount of time and memory required to assign sequences to OTUs is a legitimate concern. We observed that the time required to complete the OptiClust algorithm (Fig. 1C) paralleled the number of pairwise distances that were smaller than 0.03 (Table 1). To further evaluate how the speed and memory usage scaled with the number of sequences in the data set, we measured the time required and maximum RAM usage to cluster 20, 40, 60, 80, and 100% of the unique sequences from each of the natural data sets using the OptiClust algorithm (Fig. 2). Within each iteration of the algorithm, each sequence is compared to every other sequence and each comparison requires a recalculation of the confusion matrix. This would result in a worst-case algorithmic complexity on the order of *n*^3^, where *n* is the number of unique sequences. Because the algorithm needs to keep track of only the sequence pairs that are within the threshold of each other, it is likely that the implementation of the algorithm is more efficient. To empirically determine the algorithmic complexity, we fitted a power law function to the data in Fig. 2A. We observed power coefficients between 1.7 and 2.5 for the marine and human data sets, respectively. The algorithm requires storing a matrix that contains the pairs of sequences that are close to each other as well as a matrix that indicates which sequences are clustered together. The memory required to store these matrices is on the order of *n*^2^, where *n* is the number of unique sequences. In fact, when we fitted a power law function to the data in Fig. 2B, the power coefficients were 1.9. Using the four natural community data sets, doubling the number of sequences in a data set would increase the time required to cluster the data by 4- to 8-fold and increase the RAM required by 4-fold. It is possible that future improvements to the implementation of the algorithm could improve this performance.

### The cluster splitting heuristic generates OTUs that are as good as those generated by the nonsplitting approach.

**FIG 3:**
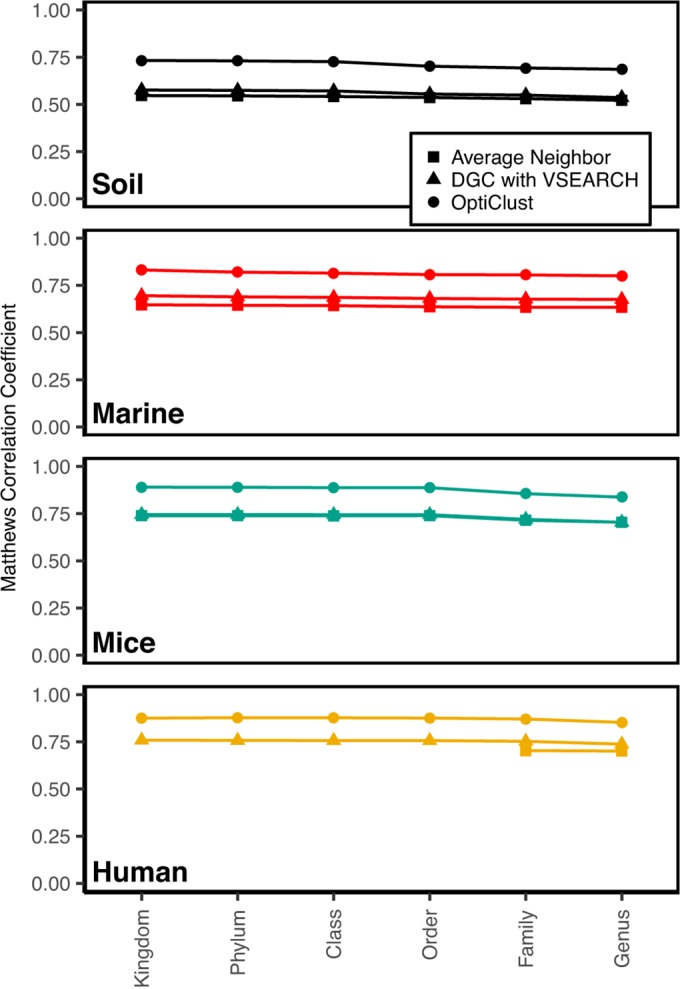
Effects of taxonomically splitting the data sets on clustering quality. The data sets were split at each taxonomic level based on their classification using a naive Bayesian classifier and clustered using average neighbor, VSEARCH-based DGC, and OptiClust.

We previously described a heuristic to accelerate OTU assignments where sequences were first classified to taxonomic groups and within each taxon sequences were assigned to OTUs using the average neighbor clustering algorithm (13). This method is similar to open-reference clustering except that in our approach all sequences are subjected to *de novo* clustering following classification, whereas in open-reference clustering only those sequences that cannot be classified are subjected to *de novo* clustering. Our cluster splitting approach accelerated the clustering and reduced the memory requirements because the number of unique sequences was effectively reduced by splitting sequences across taxonomic groups. Furthermore, because sequences in different taxonomic groups are assumed to belong to different OTUs, they are independent, which permits parallelization and additional reduction in computation time. Reduction in clustering quality is encountered in this approach if there are errors in classification or if two sequences within the desired threshold belong to different taxonomic groups. It is expected that these errors would increase as the taxonomic level goes from kingdom to genus. To characterize the clustering quality, we classified each sequence at each taxonomic level and calculated the MCC values using OptiClust, average neighbor, and DGC with VSEARCH when splitting at each taxonomic level (Fig. 3). For each method, the MCC values decreased as the taxonomic resolution increased; however, the decrease in MCC was not as large as the difference between clustering methods. As the resolution of the taxonomic levels increased, the clustering quality remained high, relative to clusters formed from the entire data set (i.e., kingdom level). The MCC values when splitting the data sets at the class and genus levels were within 98.0 and 93.0%, respectively, of the MCC values obtained from the entire data set. These decreases in MCC value resulted in the formation of as many as 4.7 and 22.5% more OTUs, respectively, than were observed from the entire data set. These errors were due to the generation of additional false negatives due to splitting similar sequences into different taxonomic groups. For the data sets included in the current analysis, the use of the cluster splitting heuristic was probably not worth the loss in clustering quality. However, as data sets become larger, it may be necessary to use the heuristic to cluster the data into OTUs.

## DISCUSSION

Myriad methods have been proposed for assigning 16S rRNA gene sequences to OTUs. Each claims improved performance based on speed, memory usage, representation of taxonomic information, and number of OTUs. Each of these metrics is subjective and does not actually indicate the quality of the clustering. This led us to propose using the MCC as a metric for assessing the quality of clustering, *post hoc*. Here, we described a new clustering method that seeks to optimize clustering based on an objective criterion that measures clustering quality in real time. In the OptiClust algorithm, clustering is driven by optimizing a metric that assesses whether any two sequences should be grouped into the same OTU. The result is clusters that are significantly more robust and is efficient in the time and memory required to cluster the sequences into OTUs. This makes it more tractable to analyze large data sets without sacrificing clustering quality, as was previously necessary using heuristic methods.

The cluster optimization procedure is dependent on the metric that is chosen for optimization. We employed the MCC because it includes the four values from a confusion matrix. Other algorithms, such as the furthest neighbor and nearest neighbor algorithms, minimize the number of FPs and FNs, respectively; however, these suffer because the numbers of FNs and FPs are not controlled, respectively (13, 16). Alternatively, one could optimize based on the sensitivity, specificity, or accuracy, which is each based on two values from the confusion matrix, or could optimize based on the F1 score, which is based on three values from the confusion matrix. Because these metrics do not balance all four parameters equally, it is likely that one parameter will dominate in the optimization procedure. For example, optimizing for sensitivity could lead to a large number of FPs. A higher number of FPs increases the number of OTUs, while a higher number of FNs collapses OTUs together. It is difficult to know which is worse since community richness and diversity are linked to the number of OTUs. In addition, increasing the number of FNs would overstate the differences between communities while increasing the number of FPs would overstate their similarity. Therefore, it is important to jointly minimize the number of FPs and FNs. With this in mind, we decided to optimize utilizing the MCC. It is possible that other metrics that balance the four parameters could be developed and employed for optimization of the clustering.

The OptiClust algorithm is relatively simple. For each sequence, it effectively asks whether the MCC value will increase if the sequence is moved to a different OTU, including creating a new OTU. If the value does not change, it remains in the current OTU. The algorithm repeats until the MCC value stabilizes. Assuming that the algorithm is seeded with each sequence in a separate OTU, it does not appear that the algorithm converges to a local optimum. Furthermore, execution of the algorithm with different random number generator seeds produces OTU assignments of consistently high quality. Future improvements to the implementation of the algorithm could provide optimization to further improve its speed and susceptibility to find a local optimum. Users are encouraged to repeat the OTU assignment several times to confirm that they have found the best OTU assignments.

Our previous MCC-based analysis of clustering algorithms indicated that the average neighbor algorithm consistently produced the best OTU assignments, with the DGC-based method using USEARCH also producing robust OTU assignments. The challenge in using the average neighbor algorithm is that it requires a large amount of RAM and is computationally demanding. This led to the development of a splitting approach that divides the clustering across distinct taxonomic groups (13). The improved performance provided by the OptiClust algorithm likely makes such splitting unnecessary for most current data sets. We have demonstrated that although the OTU assignments made at the genus level are still better than those of other methods, the quality is not as good as that found without splitting. The loss of quality is likely due to misclassification because of limitations in the clustering algorithms and reference databases. The practical significance of such small differences in clustering quality remain to be determined; however, based on the current analysis, it does appear that the number of OTUs is artificially inflated. Regardless, the best clustering quality should be pursued given the available computer resources.

The time and memory required to execute the OptiClust algorithm scaled proportionally to the number of unique sequences raised to the second power. The power for the time requirement is affected by the similarity of the sequences in the data set, with data sets containing more-similar sequences having a higher power. Also, the number of unique sequences is the basis for the amount of both time and memory required to complete the algorithm. Both the similarity of sequences and the number of unique sequences can be driven by the sequencing error, since any errors will increase the number of unique sequences and these sequences will be closely related to the perfect sequence. This underscores the importance of reducing the noise in the sequence data (7). If sequencing errors are not remediated and are relatively randomly distributed, then it is likely that the algorithm will require an unnecessary amount of time and RAM to complete.

The rapid expansion in sequencing capacity has demanded that the algorithms used to assign 16S rRNA gene sequences to OTUs be efficient while maintaining robust assignments. Although database-based approaches have been proposed to facilitate this analysis, they are limited by their limited coverage of bacterial taxonomy and by the inconsistent process used to name taxa. The ability to assign sequences to OTUs using an algorithm that optimizes clustering by directly measuring quality will significantly enhance downstream analysis. The development of the OptiClust algorithm represents a significant advance that is likely to have numerous other applications.

## MATERIALS AND METHODS

### Sequence data and processing steps.

To evaluate the OptiClust and the other algorithms, we created two synthetic sequence collections and four sequence collections generated from previously published studies. The V4 region of the 16S rRNA gene was used from all data sets because it is a popular region that can be fully sequenced with 2-fold coverage using the commonly used MiSeq sequencer from Illumina (7). The method for generating the simulated data sets followed the approach used by Kopylova et al. (34) and Schloss (36). Briefly, we randomly selected 10,000 unique V4 fragments from 16S rRNA gene sequences that were unique from the SILVA nonredundant database (38). A community with an even relative abundance profile was generated by specifying that each sequence had a frequency of 100 reads. A community with a staggered relative abundance profile was generated by specifying that the abundance of each sequence was a randomly drawn integer sampled from a uniform distribution between 1 and 200. Sequence collections collected from human feces (39), murine feces (40), soil (41), and seawater (42) were used to characterize the algorithms’ performance with natural communities. These sequence collections were all generated using paired 150- or 250-nucleotide (nt) reads of the V4 region. We reprocessed all of the reads using a common analysis pipeline that included quality score-based error correction (7), alignment against a SILVA reference database (38, 43), screening for chimeras using UCHIME (9), and classification using a naive Bayesian classifier with the RDP training set requiring an 80% confidence score (10).

### Implementation of clustering algorithms.

In addition to the OptiClust algorithm, we evaluated 10 different *de novo* clustering algorithms. These included three hierarchical algorithms, average neighbor, nearest neighbor, and furthest neighbor, which are implemented in mothur (v.1.39.0) (11). Seven heuristic methods were also used, including abundance-based greedy clustering (AGC) and distance-based greedy clustering (DGC) as implemented in USEARCH (v.6.1) (17) and VSEARCH (v.2.3.3) (18), OTUCLUST (v.0.1) (19), and Sumaclust (v.1.0.20) and Swarm (v.2.1.9) (20). With the exception of Swarm, each of these methods uses distance-based thresholds to report OTU assignments. We also evaluated the ability of OptiClust to optimize to metrics other than MCC. These included accuracy, F1 score, negative predictive value, positive predictive value, false discovery rate, sensitivity, specificity, the sum of TPs and TNs, the sum of FPs and FNs, and the number of FNs, FPs, TNs, and TPs (see Fig. S1 and Table S1 in the supplemental material).

### Benchmarking.

We evaluated the quality of the sequence clustering, the reproducibility of the clustering, the speed of clustering, and the amount of memory required to complete the clustering. To assess the quality of the clusters generated by each method, we counted the cells within a confusion matrix that indicated how well the clusterings represented the distances between the pair of sequences (13). Pairs of sequences that were in the same OTU and had a distance less than 3% were true positives (TPs), those that were in different OTUs and had a distance greater than 3% were true negatives (TNs), those that were in the same OTU and had a distance greater than 3% were false positives (FPs), and those that were in different OTUs and had a distance less than 3% were false negatives (FNs). To synthesize the matrix into a single metric, we used the Matthews correlation coefficient using the sens.spec command in mothur using the following equation:

$$\text{MCC}=\frac{\text{TP}\times \text{TN}-\text{FP}\times \text{FN}}{\sqrt{\left(\text{TP}+\text{FP}\right)\left(\text{TP}+\text{FN}\right)\left(\text{TN}+\text{FP}\right)\left(\text{TN}+\text{FN}\right)}}$$

To assess the reproducibility of the algorithms, we randomized the starting order of each sequence collection 10 times and ran each algorithm on each randomized collection. We then measured the MCC for each randomization and quantified their percent coefficient of variation (% CV; 100 times the ratio of the standard deviation to the mean).

To assess how the memory and time requirements scaled with the number of sequences included in each sequence collection, we randomly subsampled 20, 40, 60, or 80% of the unique sequences in each collection. We obtained 10 subsamples at each depth for each data set and ran each collection (*n =* 50 = 5 sequencing depths × 10 replicates) through each of the algorithms. We used the timeout script to quantify the maximum RAM used and the amount of time required to process each sequence collection (https://github.com/pshved/timeout). We limited each algorithm to 45 GB of RAM and 50 h using a single processor.

### Data and code availability.

The workflow utilized commands in GNU make (v.3.81), GNU bash (v.4.1.2), mothur (v.1.39.0) (11), and R (v.3.3.2) (44). Within R, we utilized the wesanderson (v.0.3.2) (45), dplyr (v.0.5.0) (46), tidyr (v.0.6.0) (47), cowplot (v.0.6.3) (48), and ggplot2 (v.2.2.0.9000) (49) packages. A reproducible version of the manuscript and analysis is available at https://github.com/SchlossLab/Westcott_OptiClust_mSphere_2017.
