# Supplementary material for: OptiClust, an Improved Method for Assigning Amplicon-Based Sequence Data to Operational Taxonomic Units
Source: mSphere. 2017 Mar 8;2(2):e00073-17. doi: 10.1128/mSphereDirect.00073-17 (PMC5343174; doi:10.1128/mSphereDirect.00073-17)
Supplement: TEXT S1 [file sph002172247s4.pdf]

Supplemental Text

To provide a detailed demonstration of how the OptiClust algorithm works consider a relatively simple example where there are 50 sequences. After aligning the sequences and calculating the pairwise distances between the sequences there are 15 pairs of sequences with a distance below the desired threshold of 0.03:

Seq. 1	Seq. 2	Distance
D	B	0.024
F	E	0.028
G	C	0.028
H	A	0.027
I	B	0.016
I	D	0.024
J	A	0.028
J	H	0.024
N	M	0.024
O	L	0.024
P	K	0.016
P	L	0.027
P	O	0.027
Q	E	0.024
Q	F	0.028

The other 1210 distances were larger than 0.03 and are not needed for the analysis because they are taken to be true negatives. For example, the distance between sequences A and B is larger than 0.03. So, if they are in different OTUs then that would be a true negative (TN) and if they are in the same OTU then that would be a false positive (FP). Alternatively, because sequences B and D are closer to each other than 0.03 (i.e. 0.024) if they are in separate OTUs then that would be a false negative (FN) and if they are in the same OTU then that would be a true positive (TP). It is important to note that the algorithm assumes that there are no duplicate sequences and the actual abundance is saved elsewhere to be substituted later when counting the frequency distribution of each OTU across the samples included in the analysis.

The algorithm starts by seeding sequences either into individual OTUs or into a single OTU. As demonstrated in Figure 1, seeding the sequences into randomly ordered individual OTUs generates better results and is faster than starting with a single OTU. Among the 15 pairwise distances that are smaller than 0.03, there are 17 sequences that are labeled A through Q. A separate pool is created for the 33 other sequences that are not within 0.03 of any other sequence and are thus to be placed into 33 separate OTUs. In the diagrams below, this pool is designated as "…". Having seeded the initial OTUs there are 0 TPs, 1210 TNs, 0 FPs, and 15 FNs. Initially the number of FNs corresponds to the number of distances less than 0.03, the number of TNs is the number of total distances (i.e. 1225) minus the number of distances less than 0.03. The number of TPs, TNs, FPs, and FNs should sum to the total number of distances. The resulting Matthew's Correlation Coefficient (MCC) is 0.00. The algorithm next goes through each sequence sequentially to determine whether the MCC

value would be increased by removing it from its current OTU to join other sequences in a new OTU or to create its own OTU.

The demonstration of the algorithm starts with sequence A. Notice that it is within 0.03 of sequences H and J. There are three options: sequence A could remain as its own OTU, it could join with sequence H, or it could join with sequence J.

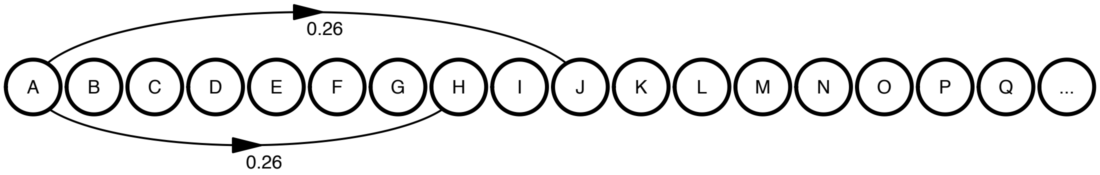

If it stays as its own OTU, the MCC value would remain 0.00. If it joined with sequences H or J the number of TPs would increase by 1 and the number of FNs would decrease by 1. In either case the MCC would be 0.26. In this case, joining H or J would result in an improved MCC and so the algorithm randomly selects which sequences to join. For this demonstration, it will form a new OTU with sequence H. This results in 1 TPs, 1210 TNs, 0 FPs, 14 FNs, and an MCC of 0.26.

Sequence B is processed by the same process as sequence A. Sequence B is within 0.03 of sequences D and I. Again, there are three options: sequence B could remain as its own OTU, it could join with sequence D, or it could join with sequence I.

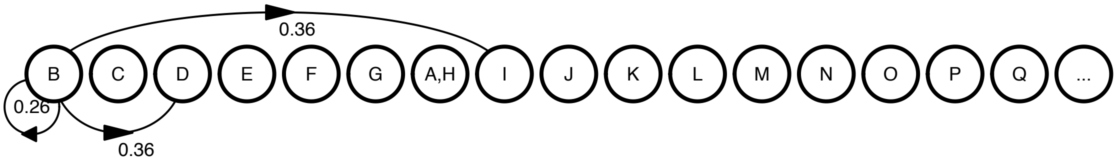

If it remains as its own OTU, the MCC value would remain 0.26. If it joined with sequences D or I the number of TPs would increase by 1 and the number of FNs would decrease by 1. In either case the MCC would be 0.36. Joining D or I would result in an improved MCC and so the algorithm randomly selects which sequences to join. For this demonstration it will form a new OTU with sequence D. This results in 2 TPs, 1210 TNs, 0 FPs, 13 FNs, and an MCC of 0.36.

Sequence C is within 0.03 of sequence G creating two options: remain as its own OTU or join with sequence G.

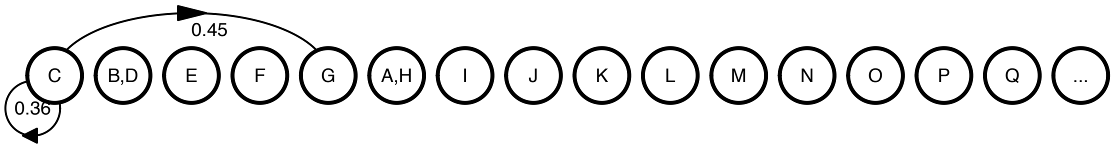

If it remains in its own OTU, the MCC value would remain 0.36. If it joined with sequence G the number of TPs would increase by 1 and the number of FNs would decrease by 1 resulting in an MCC value of 0.45. Because of the improved MCC value, sequence C joins with sequence G to form a new OTU. This results in 3 TPs, 1210 TNs, 0 FPs, 12 FNs, and an MCC of 0.45.

For sequence D the algorithm gets more complicated since sequence D is already in an OTU with sequence B. As seen when considering sequence B, sequence D is within 0.03 of sequence B and it is also similar to sequence I. Now there are three options: sequence D could remain with sequence B in an OTU, it could leave that OTU and join with sequence I, or it could form a new OTU where it is the sole member.

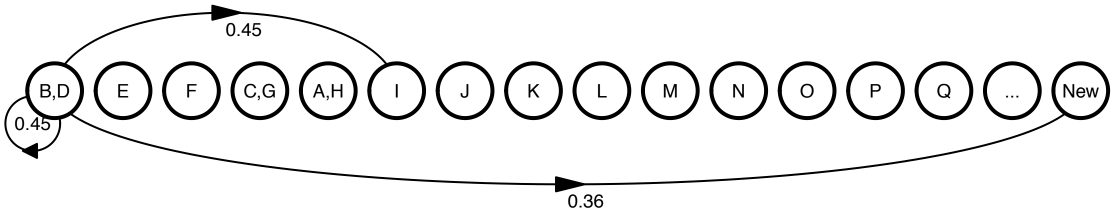

If sequence D remains with B, the MCC value would remain 0.45. If it joined with sequence I the number of TPs and FNs would stay constant resulting in an MCC value of 0.51. If it formed a new OTU by itself, the number of TPs would decrease by one and the number of FNs would increase by 1 resulting in an MCC value of 0.36. Because the MCC values for staying in the OTU with B or leaving the OTU to join the OTU with I are the same, the algorithm would again randomly chose between the two options. For demonstration purposes, sequence D will remain in its OTU with sequence B. This results in no changes in the four parameters or the MCC value.

For sequence E, the same type of options are available as when sequences A and B were processed. Sequence E could remain on its own or it could join with sequences F or Q.

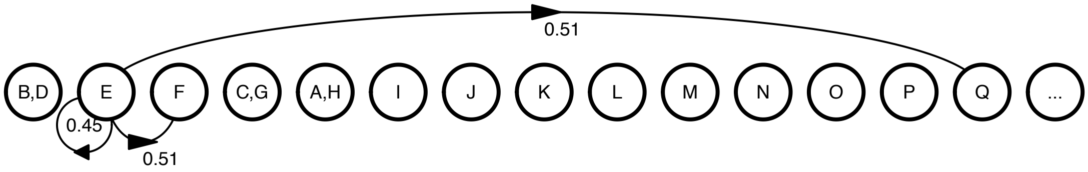

Similar to the earlier cases, the MCC value for joining another sequence is larger than staying on its own. Because the MCC values for joining F or Q are the same, the algorithm randomly selects which sequences to join. For this demonstration sequence E will form a new OTU with sequence F. This results in 4 TPs, 1210 TNs, 0 FPs, 11 FNs, and an MCC of 0.51.

For sequence F, the steps taken by the algorithm are the same as earlier for sequence D. Here, sequence F could remain in an OTU with sequence E, it could leave and form an OTU with sequence Q, or it could form a new OTU on its own.

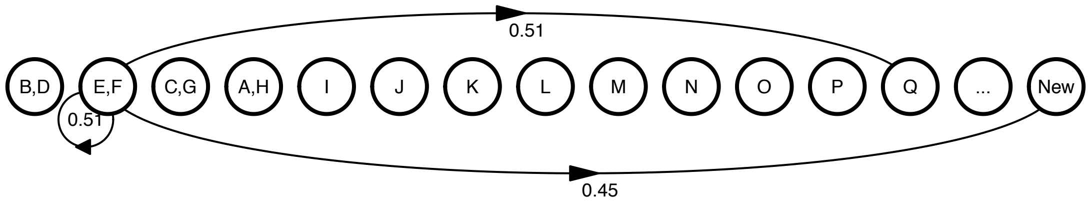

Again, the MCC value for sequence F remaining with sequence E is the same as for leaving to form an OTU with sequence Q. Both options are superior to leaving to form a new OTU on its own. The algorithm randomly chooses between the two options. For demonstration purposes, sequence F will remain in its OTU with sequence E. This results in no changes in the four parameters or the MCC value.

The decisions for sequence G are similar to sequence F, with the exception that the only choices are to stay in an OTU with another sequence (C) or to form a new OTU on its own.

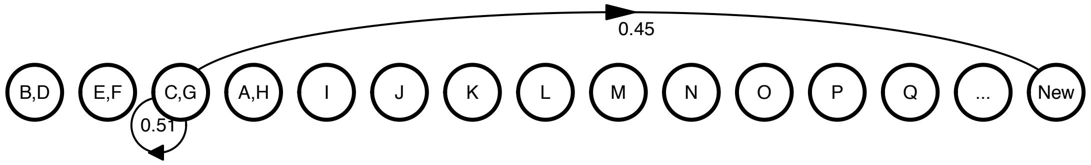

Leaving to form a new OTU results in a lower MCC value and so the algorithm leaves sequence G with sequence C. This results in no changes in the four parameters or the MCC value.

For sequence H the steps taken are the same as seen earlier for sequence B.

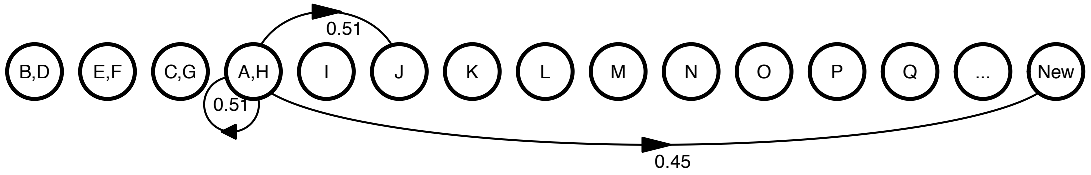

For demonstration purposes sequence H will remain in its OTU with sequence A.

Moving to sequence I, the process is similar to what was done earlier with sequences A and B. The only difference is that because sequence I is similar to both sequences B and D the increase in TP and decrease in FN will be double.

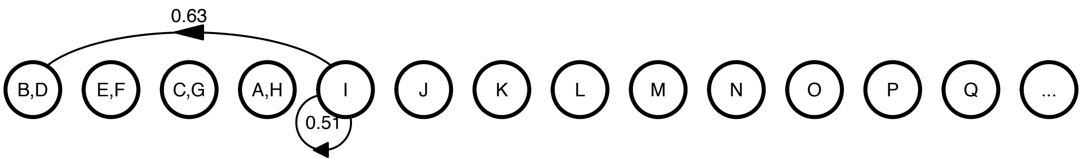

If sequence I remains in an OTU by itself, the MCC value will be 0.51. If it joined the OTU with sequences B and D, then the number of TPs would increase by 2 and the number of FNs would decrease by 2 resulting in an improved MCC value of 0.63. This is the choice that is taken resulting in 6 TPs, 1210 TNs, 0 FPs, and 9 FNs.

Processing sequence J is the same as sequence I since sequence J is close to both A and H.

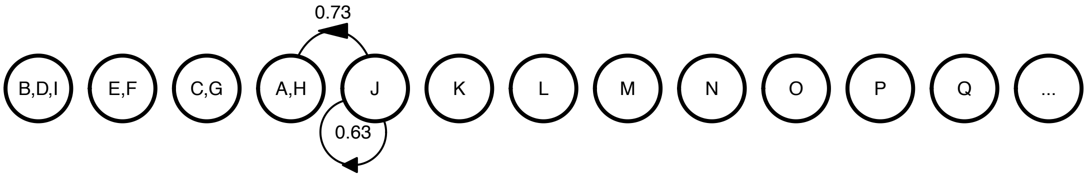

Again, by joining the OTU containing sequences A and H the number of TPs increases by 2 and the number of FNs decreases by 2 resulting in 8 TPs, 1210 TNs, 0 FPs, 7 FNs, and an improved MCC value of 0.73.

Processing sequence K is the same as for sequence A.

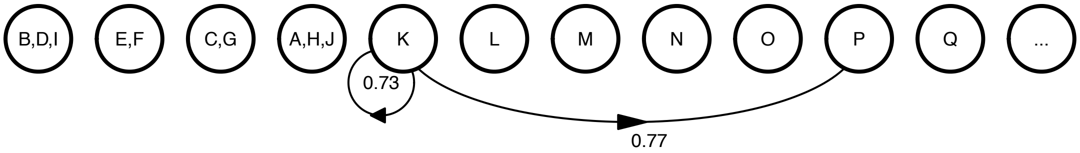

Merging sequences K and P into the same OTU results in 9 TPs, 1210 TNs, 0 FPs, 6 FNs, and an improved MCC value of 0.77.

Processing sequence L presents a more complicated set of decisions. Again, there are three choices. Because sequence L is similar to sequences O and P it could form an OTU with sequence O or with the OTU containing sequences K and P.

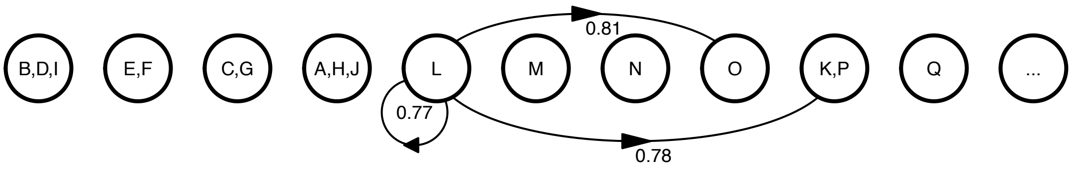

If sequence L remains on its own, the MCC value would remain 0.77. If it joined with sequence O the number of TPs would increase by 1 and the number of FNs would decrease by 1 resulting in an MCC value of 0.81. The subtlety of this step is found in when considering the possibility of sequence L joining an OTU with sequences K and P. It would increase the number of TPs by one and decrease the number of FN by one and by joining with sequence P; however, because O is not close to K the number of TNs would decrease by one and the number of FPs would increase by one. This would result in an MCC value of 0.78. Of the three options forming an OTU with sequences L and O provides the maximal MCC value. This results in 10 TPs, 1210 TNs, 0 FPs, 5 FNs, and an improved MCC value of 0.81.

The steps for processing sequence M is the same as earlier for sequence C.

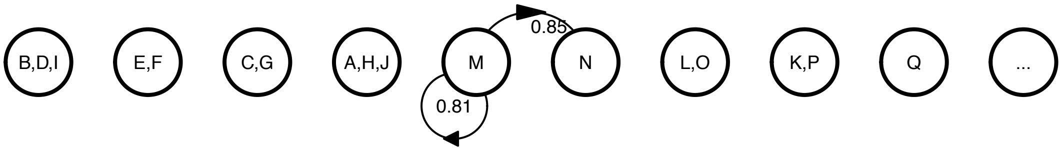

Merging sequences M and N into the same OTU results in 11 TPs, 1210 TNs, 0 FPs, 4 FNs, and an improved MCC value of 0.85.

Moving on to sequence N, the two options are to stay in an OTU with sequence M or to spit off and form a new OTU on its own.

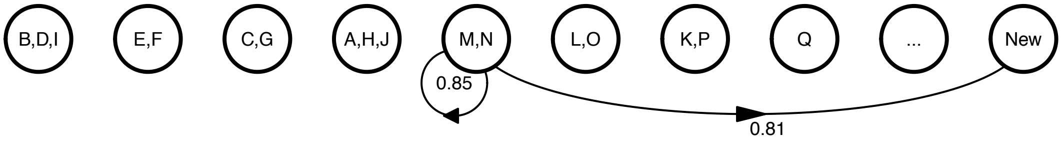

Remaining in an OTU with sequence M provides the larger MCC value and so the OTU memberships do not change.

Processing of sequence O presents three options that have been explored before. Sequence O can stay in its OTU with sequence L, it can join the OTU with sequences K and P, or it can form a new OTU on its own.

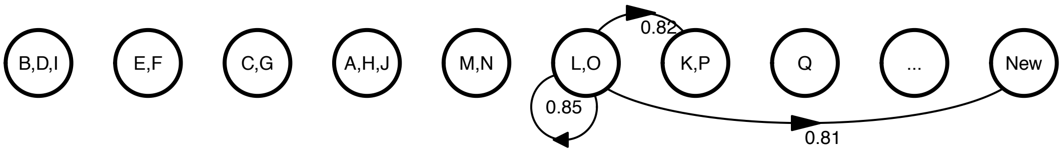

If sequence O remains in the OTU with sequence L, the MCC value would be 0.85. If it leaves that OTU to join sequences K and P in their OTU then the number of TNs would decrease by 1, but the number of FPs would increase by 1 because O is similar to P, but not to K. This would result in an MCC value of 0.82. If sequence O forms a new OTU on its own, then the number of TPs would decrease by one and the number of FNs would increase by one resulting in an MCC value of 0.81. The best option is for sequence O to remain in its OTU with sequence L.

For sequence P the steps taken are similar to those used to evaluate clusters for sequence O; however, the final decision is different. Sequence P can stay with sequence K in their OTU, it can leave to join the OTU with sequences L and O, or it can form a new OTU on its own.

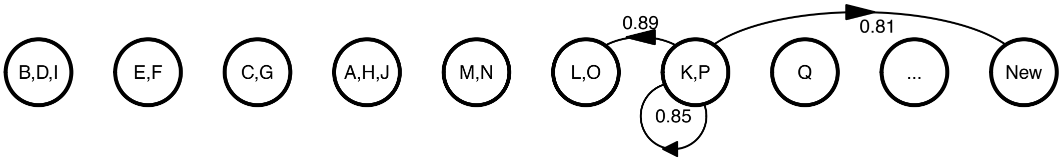

If sequence P remains in the OTU with sequence K, the MCC value would be 0.85. Alternatively, P could leave that OTU to join sequences L and O in their OTU. If P leaves its OTU with K then the number of TPs would decrease by one and the number of FNs would increase by one. By joining with L and O the number of TPs would increase by two and the number of FNs would decrease by two. The net effect would be to increase the number of TPs by 1 and decrease the number of FNs by 1. This would result in an MCC value of 0.89. If sequence P formed a new OTU on its own, then the number of TPs would decrease by one and the number of FNs would increase by one resulting in an MCC value of 0.81. The best option is for sequence P to leave its OTU with sequence K and join the OTU containing sequences L and O. The updated counts are 12 TPs, 1210 TNs, 0 FPs, 3 FNs.

To finish the first round of processing each sequence, sequence Q is processed. Sequence Q is similar to both sequences E and F. Because sequences E and F are in the same OTU, the situation is similar to processing sequence I.

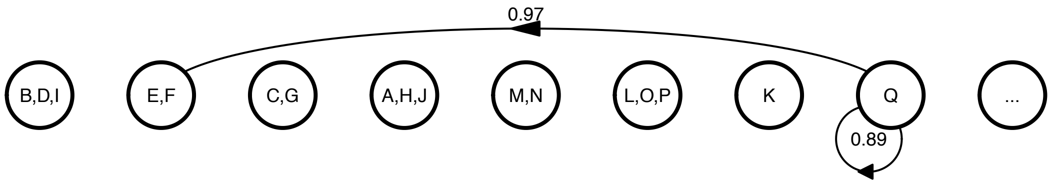

By joining the OTU containing sequences E and F the number of TPs increases by two and the number of FNs decreases by one. The updated counts are 14 TPs, 1210 TNs, 0 FPs, and 1 FNs, which result in an improved MCC value of 0.97.

Having processed each sequence, the first iteration of the algorithm is complete. The MCC value has changed from 0.00 to 0.97. Because the MCC value changed, it is necessary to re-evaluate each sequence again and re-evaluate the final MCC value to determine whether it has changed. In this case, evaluation of sequences A through J result in the same clustering pattern. When the algorithm reaches sequence K it finds that the sequence is similar to sequence P, which is in an OTU with L and O; however sequence K is not similar to L or O.

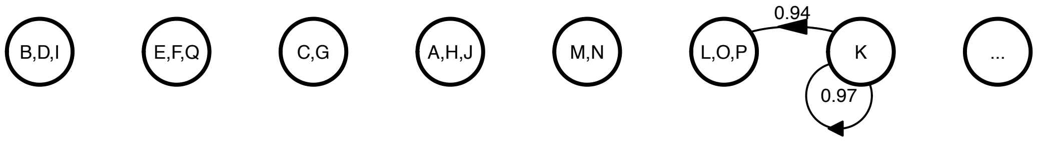

Although, sequence K is similar to sequence P, it is not similar to sequences L or O. Were sequence K to join their OTU, it would increase the number of TPs by one and decrease the number of FNs by one because of its similarity to sequence P, but it would increase the number of FPs by two and decrease the number of TNs by two because K is not similar to L or O. The end result would be a MCC value of 0.94, which is less than the MCC value of keeping sequence K on its own (i.e. 0.97).

Continuing the process for the remaining sequences, none of the sequences will move between OTUs and the MCC value does not change. At this point, the clustering has converged to the optimum MCC value of 0.97. Repeating this process using 100 different seeds for the random number generator required a median of 3 iterations (range from 2 to 4) to converge.
