# Supplementary material for: OptiClust, an Improved Method for Assigning Amplicon-Based Sequence Data to Operational Taxonomic Units
Source: mSphere. 2017 Mar 8;2(2):e00073-17. doi: 10.1128/mSphereDirect.00073-17 (PMC5343174; doi:10.1128/mSphereDirect.00073-17)
Supplement: TABLE S1 [file sph002172247st1.pdf]

Table S1. Summary of the average number of true positives, true negatives, false positives, false negatives and the resulting Matthews correlation coefficient for each of the clustering methods that were analyzed in this study for each of the six datasets. Blank values indicate that those conditions could not be completed in 50 hours with 45 GB of RAM.

Dataset	Method	True Positives	True Negatives	False Positives	False Negatives	MCC
Soil	MCC (separate OTUs)	5136712	10312528053	1247993	2555568	0.7328
Soil	TP + TN	4770846	10312976362	799684	2921434	0.7287
Soil	Accuracy	4771037	10312974895	801151	2921243	0.7286
Soil	FPs + FNs	4768761	10312976641	799405	2923519	0.7285
Soil	MCC (single OTU)	5399187	10311962308	1813738	2293093	0.7247
Soil	F1-score	5453578	10311862032	1914014	2238702	0.7244
Soil	VSEARCH (w/AGC)	5953502	10305863000	7913046	1738778	0.5760
Soil	VSEARCH (w/AGC)	6684050	10301990342	11785704	1008230	0.5603
Soil	Sumaclust	6689597	10301700186	12075860	1002683	0.5563
Soil	Average Neighbor	6746206	10300797169	12978877	946074	0.5472
Soil	USEARCH (w/DGC)	4851924	10308165644	5610402	2840356	0.5404
Soil	USEARCH (w/AGC)	5592316	10304608407	9167639	2099964	0.5244
Soil	OTUCLUST	6714016	6909706980	18444775	978264	0.4819
Soil	Negative Predictive Value	7382082	10286785610	26990436	310198	0.4534
Soil	FN	7438201	10283907614	29868432	254079	0.4386
Soil	Nearest Neighbor	1571726	10313677482	98564	6120554	0.4383
Soil	Sensitivity	7440261	10283040048	30735998	252019	0.4335
Soil	Furthest Neighbor	501819	10313776046	0	7190461	0.2553
Soil	Swarm	237705	10313770690	5356	7454575	0.1738
Marine	F1-score	7914666	2870997648	1894417	1306272	0.8317
Marine	MCC (separate OTUs)	7670655	2871231788	1660277	1550283	0.8266
Marine	MCC (single OTU)	7428998	2871482154	1409911	1791940	0.8223
Marine	FPs + FNs	7264205	2871683595	1208470	1956733	0.8214
Marine	TP + TN	7211154	2871739258	1152807	2009784	0.8206
Marine	Accuracy	7119498	2871802386	1089679	2101440	0.8178
Marine	VSEARCH (w/AGC)	8566881	2865055591	7836474	654057	0.6954
Marine	VSEARCH (w/AGC)	8867443	2862976753	9915312	353495	0.6725
Marine	Sumaclust	8867317	2862856105	10035960	353621	0.6703
Marine	USEARCH (w/DGC)	6929948	2867522405	5369660	2290990	0.6494
Marine	Average Neighbor	8914621	2861348612	11543453	306317	0.6476
Marine	USEARCH (w/AGC)	7352302	2865419824	7472241	1868636	0.6274
Marine	Nearest Neighbor	3742329	2872622032	270033	5478609	0.6144
Marine	OTUCLUST	8945456	1834755673	15224413	275482	0.5965
Marine	Negative Predictive Value	9131188	2853099098	19792967	89750	0.5572
Marine	Sensitivity	9136046	2852367594	20524471	84892	0.5504
Marine	FN	9143826	2852250720	20641345	77112	0.5498
Marine	Furthest Neighbor	620264	2872892065	0	8600674	0.2589
Marine	Swarm	149810	2872888019	4046	9071128	0.1256
Mice	MCC (separate OTUs)	4829770	520373752	491600	692559	0.8898
Mice	F1-score	4850792	520347542	517810	671537	0.8897
Mice	FPs + FNs	4771312	520439019	426333	751016	0.8895
Mice	TP + TN	4770937	520439150	426202	751392	0.8894
Mice	Accuracy	4769575	520440407	424945	752754	0.8894
Mice	MCC (single OTU)	4879087	518832093	2033259	643242	0.7873
Mice	VSEARCH (w/AGC)	5100191	517549332	3316020	422138	0.7450
Mice	USEARCH (w/DGC)	4905962	517883072	2982280	616367	0.7402
Mice	Average Neighbor	5326219	516873116	3992236	196110	0.7393
Mice	Sumaclust	5317976	516662498	4202854	204353	0.7301
Mice	VSEARCH (w/AGC)	5322737	516622070	4243282	199592	0.7290
Mice	USEARCH (w/AGC)	5243162	516749770	4115582	279167	0.7260
Mice	Negative Predictive Value	5426962	515767988	5097364	95366	0.7082
Mice	Sensitivity	5439118	515571271	5294081	83211	0.7028
Mice	FN	5451490	515436798	5428554	70839	0.6996
Mice	OTUCLUST	5297141	469202414	5087312	225188	0.6953
Mice	Nearest Neighbor	1482308	520843906	21446	4040021	0.5122
Mice	Swarm	1237099	520494387	370965	4285230	0.4123
Mice	Furthest Neighbor	665512	520865352	0	4856816	0.3455
Human	F1-score	26073348	7321213423	2922678	4270391	0.8785
Human	MCC (separate OTUs)	25861724	7321423671	2712430	4482015	0.8778
Human	FPs + FNs	25512998	7321813377	2322724	4830740	0.8774
Human	MCC (single OTU)	25680854	7321538561	2597540	4662885	0.8762
Human	TP + TN	25399667	7321870325	2265776	4944072	0.8762
Human	Accuracy	25352243	7321910658	2225443	4991496	0.8759
Human	VSEARCH (w/AGC)	26845545	7309824393	14311708	3498194	0.7585
Human	VSEARCH (w/AGC)	28561130	7301551637	22584464	1782609	0.7237
Human	Negative Predictive Value	29719012	7292417394	31718707	624727	0.6867
Human	Sensitivity	29818923	7288099634	36036467	524816	0.6657
Human	FN	29888157	7287209148	36926953	455582	0.6623
Human	Nearest Neighbor	8062473	7323981460	154641	22281266	0.5097
Human	Swarm	4358052	7323725163	410938	25985688	0.3615
Human	Furthest Neighbor	2637845	7324136101	0	27705894	0.2942
Human	Average Neighbor					
Human	OTUCLUST					
Human	Sumaclust					
Human	USEARCH (w/AGC)					
Human	USEARCH (w/DGC)					
Even	MCC (separate OTUs)	17174	66762945	2465	5319	0.8171
Even	F1-score	17586	66762397	3013	4907	0.8169
Even	MCC (single OTU)	17174	66762930	2480	5319	0.8168
Even	TP + TN	16378	66763755	1655	6115	0.8132
Even	Accuracy	16349	66763784	1626	6144	0.8130
Even	FPs + FNs	16312	66763795	1615	6181	0.8123
Even	Average Neighbor	15607	66763393	2017	6886	0.7838
Even	Nearest Neighbor	12448	66764886	524	10046	0.7287
Even	USEARCH (w/DGC)	12950	66762468	2942	9544	0.6847
Even	Negative Predictive Value	20746	66744250	21160	1747	0.6768
Even	VSEARCH (w/AGC)	12340	66762579	2831	10153	0.6679
Even	VSEARCH (w/AGC)	12340	66762579	2831	10153	0.6679
Even	Furthest Neighbor	9434	66765410	0	13059	0.6475
Even	Sumaclust	12559	66761243	4167	9934	0.6474
Even	FN	21047	66738879	26531	1446	0.6436
Even	Sensitivity	21106	66735974	29436	1387	0.6299
Even	USEARCH (w/AGC)	11260	66761521	3889	11233	0.6096
Even	OTUCLUST	16844	66725346	18107	5649	0.6007
Even	Swarm	520	66765401	9	21973	0.1507
Staggered	MCC (separate OTUs)	17069	66763106	2304	5424	0.8176
Staggered	F1-score	17584	66762432	2978	4909	0.8176
Staggered	MCC (single OTU)	17200	66762827	2583	5293	0.8154
Staggered	Accuracy	16337	66763813	1597	6156	0.8134
Staggered	FPs + FNs	16313	66763811	1599	6180	0.8127
Staggered	TP + TN	16319	66763779	1631	6174	0.8121
Staggered	Average Neighbor	16142	66762854	2556	6351	0.7871
Staggered	Nearest Neighbor	12413	66764886	524	10080	0.7276
Staggered	Sumaclust	15377	66760390	5020	7116	0.7178
Staggered	VSEARCH (w/AGC)	15095	66760823	4587	7398	0.7173
Staggered	USEARCH (w/AGC)	14630	66761174	4236	7863	0.7101
Staggered	VSEARCH (w/AGC)	13567	66762304	3106	8926	0.7005
Staggered	USEARCH (w/DGC)	13244	66762537	2873	9250	0.6955
Staggered	Negative Predictive Value	20724	66744588	20822	1769	0.6783
Staggered	Furthest Neighbor	9575	66765410	0	12918	0.6524
Staggered	FN	21018	66738538	26872	1475	0.6413
Staggered	Sensitivity	20981	66737899	27511	1512	0.6361
Staggered	OTUCLUST	15625	66371336	15527	6868	0.5903
Staggered	Swarm	443	66765408	2	22050	0.1400
